# Subpleural ground glass opacities diagnosed by bronchoscopic brush cytology with manual mapping navigation: A case report

**DOI:** 10.1097/MD.0000000000025515

**Published:** 2021-04-23

**Authors:** Lei Zhang, Guiqi Wang

**Affiliations:** Department of Endoscopy, National Cancer Center/National Clinical Research Center for Cancer/Cancer Hospital, Chinese Academy of Medical Sciences and Peking Union Medical College, Beijing, China.

**Keywords:** bronchoscopic brushing, bronchoscopy, case report, ground-glass opacities, manual mapping navigation, peripheral pulmonary lesions

## Abstract

**Rationale:**

Ground-glass opacity nodules (GGNs) are a computed tomography (CT) finding suggestive of lung cancer. Conventional bronchoscopy with brush cytology is a simple diagnostic modality but has a low diagnostic yield for peripheral lesions, especially peripheral GGNs. Therefore, maximizing the detection rate of bronchoscopic brushings should be a key objective. We report a case of a subpleural ground glass opacity (GGO) with a cytological diagnosis of adenocarcinoma by bronchoscopic brushing guided by manual mapping navigation.

**Patient concerns:**

A 46-year-old man was hospitalized for GGO in the right lung. Follow-up CT revealed a subpleural nodule sized 1.2 cm × 0.9 cm in the superior segment of the right lower lobe.

**Diagnoses:**

CT findings of the patient's nodule were suggestive of malignancy.

**Interventions:**

The patient underwent conventional bronchoscopy combined with brushing guided by manual mapping navigation, with subsequent cytological diagnosis of adenocarcinoma. The patient then underwent right lower lobectomy with mediastinal lymph node dissection.

**Outcomes:**

There were no postoperative complications. Postoperative pathological examination showed lung adenocarcinoma with lepidic and acinar growth without visceral pleural invasion (pT1aN0M0, IA1).

**Lessons:**

Exfoliated cells present in peripheral GGNs are rarely detected on brush sampling. However, use of a manual mapping navigation system may help increase the sensitivity of conventional bronchoscopic brushing for the diagnosis of peripheral pulmonary lesions.

## 1 Introduction

Ground glass opacity nodules (GGNs) are a computed tomography (CT) finding suggestive of lung cancer.^[1]^ The bronchoscopic brush cytology is a simple and non-invasive technique through the natural respiratory cavity, producing decisive diagnostic yields irrespective of the site and type of tumor.^[2,3]^ However, conventional bronchoscopy with brushing is less sensitive (as low as 16%) for diagnosing peripheral pulmonary lesions (PPLs), especially for peripheral GGNs.^[4]^ Maximizing the detection rate of bronchoscopic brushings should therefore be a key objective. Although advanced technologies and innovations such as C-arm fluoroscopy, electromagnetic navigation bronchoscopy, radical endobronchial ultrasound, and virtual bronchoscopy navigation can significantly improve the sensitivity of bronchoscopy brush cytology of peripheral nodules, they are expensive, time consuming, require special equipment and experienced bronchologists, or involve radiation exposure.^[5–8]^

Starting in 2010, we used a manual mapping method to guide bronchoscope insertion for brushing. This method determines the route with a series of bronchial opening sketches and marks the leading bronchus at every bifurcation point based on thin-section CT.^[4]^ This method improved the sensitivity of conventional brushing of malignant PPLs. In combination with the mapping method, the sensitivity of conventional brushing with mapping increased from 17.0% to 31.5%. After that, we used manual mapping for routine bronchoscopy with brushing. Herein, we present a case of a subpleural ground glass opacity (GGO) with a cytological diagnosis of adenocarcinoma by bronchoscopic brushing guided by manual mapping navigation.

## 2 Case presentation

**Figure 1:**
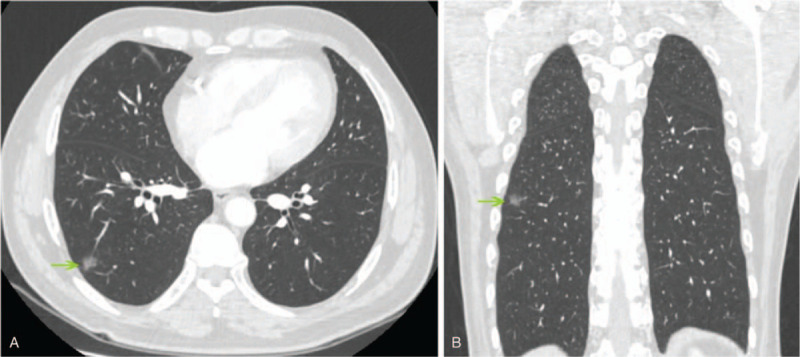
Subpleural 1.2-cm ground-glass opacity lesion (arrow) in the superior segment of the right lung lower lobe observed on chest CT imaging, with axial (A) and coronal (B) images. CT = computed tomography.

A GGO nodule was identified in the right lower lobe of a 46-year-old man during incidental CT screening performed 5 months before his visit to our hospital. Before admission, the patient underwent additional CT screening, which indicated no significant changes in the lesion. Chest CT imaging revealed a 12 mm × 9-mm solitary part-solid nodule in the superior segment of the right lower lobe of the lung (Fig. 1). The patient had comorbidities of atherosclerotic coronary heart disease, with subsequent stent implantation, but denied any other medical history. Lung function was evaluated through formal spirometry with a forced expiratory volume in the first second (FEV1) of 3.08 L (predicted 96.8%), a forced vital capacity (FVC) of 3.28 L (predicted 97%), and an FEV1/FVC ratio of 93.9%. There was no significant finding on laboratory and physical examination. The findings for the nodule were suggestive of malignancy, but the patient refused to undergo a CT-guided biopsy.

**Figure 2:**
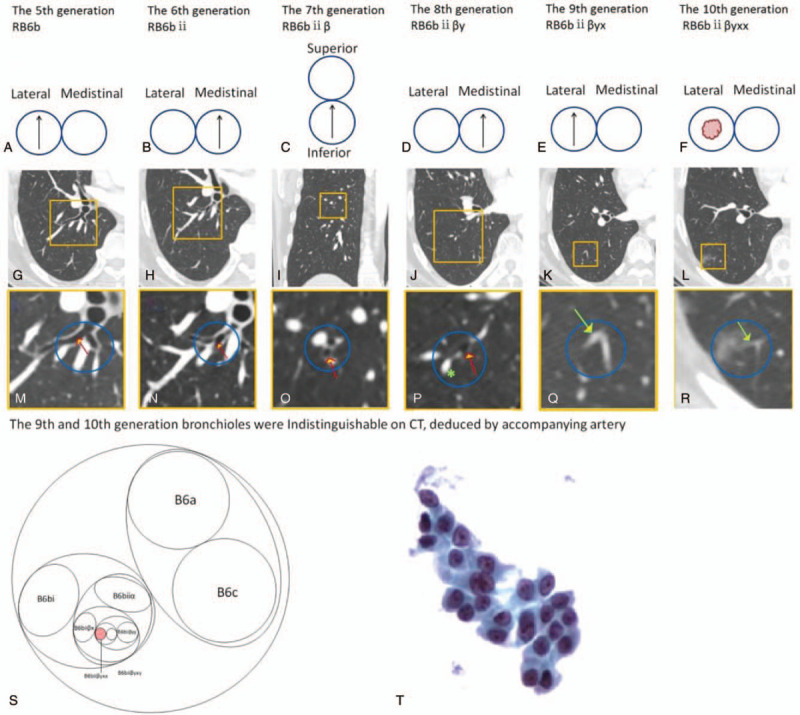
Map showing the leading bronchus of the 5th (RB6) to the 10th generation that targeted the lesion with a relative spatial position. (A–E) Bronchial opening map of the 5th to the 9th generations with the leading bronchus at the relative position (the black arrow marks the leading bronchus along the route). (F) Lesion located distal to the leading bronchus of the 10th generation of the superior segment of the right lung lower lobe. (G–L) Corresponding computed tomography images of (A–F). (M–R) Partially enlarged views of (G–L). (S) Schematic diagram of the bronchial opening of the 5th to the 10th generation. (T) Cytopathologic result of adenocarcinoma cells (Papanicolaou stain, ×40).

**Figure 3:**
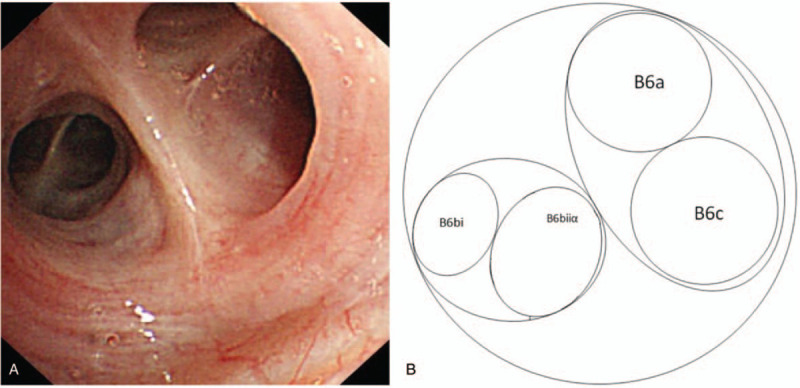
Bronchoscopic images and schematic diagram of the bronchial opening of the superior segment of the right lung lower lobe.

Before the operation, conventional bronchoscopy was performed. We manually constructed the route to the lesion, involving tracing the bronchial branch status by continuous rolling of thin-section CT images, then recording them with a series of bronchial opening sketches, marking the leading bronchus at every bifurcation point (Fig. 2). According to the results shown in the navigation map, the bronchoscope was inserted into the subsegmental bronchial lumen and the brush was extended from B6b-ii toward the target (Fig. 3). A subsequent cytological analysis revealed the presence of adenocarcinoma cells.

Considering that the exfoliated cancer cells were found in the bronchoscope brush, the surgeon decided to perform lobectomy involving mediastinal lymph node dissection directly. Briefly, the patient was placed in the left lateral decubitus position with the arms extended at an angle of 90°. General anesthesia was induced, and intubation was performed through a double-lumen endobronchial tube. An incision, which was approximately 3-cm long, was performed in the fourth intercostal space, in the anterior position. First, we performed right lower lobectomy. Moreover, examination of a frozen section confirmed the diagnosis of a lung adenocarcinoma with an acinar component. Subsequent mediastinal lymphadenectomy was performed to remove lymph nodes from the 2nd, 4th, 7th, and 10th groups.

Postoperative duration of hospitalization was 5 days. There were no postoperative complications. Postoperative pathological examination showed an invasive adenocarcinoma carcinoma with lepidic and acinar growth without visceral pleura invasion (pT1aN0M0, IA1).

## 3 Discussion

Herein, we present an unusual case of a subpleural GGO with a cytological diagnosis of adenocarcinoma obtained by bronchoscopic brushing. Generally, GGO observed on a chest CT is considered a lepidic component suggestive of adenocarcinoma in situ (AIS), minimally invasive adenocarcinoma (MIA), or lepidic adenocarcinoma. These findings are correlated with a favorable prognosis after surgical resection.^[9]^ This is mainly because the tumor cells in these situations are confined to the alveolar cavity without infiltrating into the lung interstitial tissues or without shedding from the alveoli for dissemination through the airways.^[10]^ However, there is evidence that other more invasive, non-lepidic growth patterns of adenocarcinoma, such as acinar and papillary patterns, have appeared as GGNs.^[11,12]^ Sun et al^[9]^ reported GGNs (nodule diameter <20 mm and solid component diameter <50%) in a group of 408 patients, among which 22.8% (93 cases) were identified as an invasive adenocarcinoma, including 12 cases of papillary adenocarcinoma and 1 case of micropapillary adenocarcinoma. Moreover, CT images of pure GGOs also still show acinar, papillary, and lepidic components in the tumor tissues. In these cases, exfoliated cells that shed from the alveoli may spread along the airway and disseminate; therefore, as seen in this case, it is possible to obtain a cytological diagnosis of peripheral GGO through bronchoscopic brushing, but results from a large case series are required for confirmation.

Many surgeons choose sublobar resection (wedge resection or segmentectomy) for curative treatment of patients with GGNs together with a favorable prognosis.^[9]^ However, the surgeons may face a dilemma when postoperative pathology suggests non-lepidic growth patterns of adenocarcinoma, such as acinar and papillary patterns, wherein sublobar resection may not be the best treatment of choice. Therefore, it is of interest to evaluate tumor characteristics through various diagnostic modalities preoperation. In this case, a cytological diagnosis of adenocarcinoma was obtained by bronchoscopic brushing, indicating that exfoliated cancer cells had disseminated through the airway. These findings are suggestive of an invasive adenocarcinoma. Therefore, sublobar resection was not enough, and lobectomy with mediastinal lymph node dissection was required. This is one of the interesting parts of using the preoperative bronchoscope brush in this case. However, whether or not routine bronchoscopy with brushing and selection of certain patients with peripheral GGOs are required should be unraveled in further research.

Conventional bronchoscopy with brush cytology is a simple and specific diagnostic modality, but it has a low diagnostic yield regarding the detection of peripheral lesions.^[2,13,14]^ One factor that accounts for the lower yield of conventional bronchoscopy brush is that the selection of bronchial routes to the lesion based solely on CT scans is likely to be inaccurate beyond the third- or fourth-generation bronchus and that bronchoscopes typically will not be able to reach the peripheral lung lesions. We previously reported through a retrospective cohort study that the sensitivity of conventional bronchoscopic brushing was 17% for malignant PPLs. For lesion sized <3 cm, the sensitivity of brushing as a diagnostic modality was as low as 11.1%.^[4]^ Currently, increase in the diagnostic sensitivity is possible when bronchoscopy is combined with navigation and imaging techniques such as radial-probe endobronchial ultrasound (EBUS) or navigational bronchoscopy.^[6,8,15]^ However, these advanced bronchoscopy techniques are expensive and not feasible for implementation in every institution.^[16,17]^

This representative case was cytologically diagnosed using bronchoscopic brushing guided by manual mapping. As described previously, the principle of the manual mapping method is the translation of thin-section CT information into an endoscopic route.^[4]^ Using this mapping method, the generated two-dimensional map provides information regarding the actual relative position bronchoscope and allows the bronchoscopist to recognize the route easily, thereby increasing the chance of reaching the peripheral lesions. With the use of the mapping method, the sensitivity for detecting malignant PPLs using brushing can increase from 17.0% to 31.5%. In addition, the mapping method is especially suitable for cases with larger, solid-appearing lesions, cut-off signs at the targeted bronchus, lesions nearer to the lobar bronchial opening, and clear accessibility. However, the nodule of the patient did not have these characteristics. Given the anatomy and pathologic features of the present case, the positive preoperative cytology finding may be explained by the fact that exfoliated malignant cells were probably detected using brushing of the bronchi/bronchioles, which, otherwise, cannot be detected. Therefore, a hand-drawn “bronchial map” based on CT images benefited the pulmonologist, who could then perform precise brushing.

## 4 Conclusions

This case provides evidence for the presence of peripheral GGOs diagnosed using brush cytology. Although further studies are required to generalize our findings, the manual mapping method used in this case may help improve the sensitivity and diagnostic yield when used in combination with bronchoscopy brushing in patients with findings suggestive of peripheral lung cancer. This modality can be used in most institutions that cannot offer advanced bronchoscopy techniques.

## Author contributions

**Conceptualization:** Lei Zhang, Guiqi Wang.

**Data curation:** Lei Zhang.

**Funding acquisition:** Lei Zhang.

**Supervision:** Guiqi Wang.

**Writing – original draft:** Lei Zhang.

**Writing – review & editing:** Guiqi Wang.

## References

[1] Gao JW Rizzo S Ma LH. Pulmonary ground-glass opacity: computed tomography features, histopathology and molecular pathology. Transl Lung Cancer Res 2017;6:68–75. 28331826 10.21037/tlcr.2017.01.02 PMC5344841

[2] Smith GH Warrack AJ. An evaluation of brush biopsy in the diagnosis of peripheral pulmonary lesions. Thorax 1972;27:631–5. 4343445 10.1136/thx.27.5.631 PMC470575

[3] Roth K Hardie JA Andreassen AH. Predictors of diagnostic yield in bronchoscopy: a retrospective cohort study comparing different combinations of sampling techniques. BMC Pulm Med 2008;8:02 doi:10.1186/1471-2466-8-2. 10.1186/1471-2466-8-2 PMC2267157 18221551

[4] Zhang L Tong R Wang J. Improvements to bronchoscopic brushing with a manual mapping method: a three-year experience of 1143 cases. Thorac Cancer 2016;7:72–9. 26816541 10.1111/1759-7714.12279 PMC4718127

[5] Yu KL Tsai TH Ho CC. The value of radial endobronchial ultrasound-guided bronchial brushing in peripheral non-squamous non-small cell lung cancer. Sci Rep 2018;8:5837 doi: 10.1038/s41598-018-24300-7. 29643378 10.1038/s41598-018-24300-7 PMC5895614

[6] Folch EE Labarca G Ospina-Delgado D. Sensitivity and safety of electromagnetic navigation bronchoscopy for lung cancer diagnosis: systematic review and meta-analysis. Chest 2020;158:1753–69. 32450240 10.1016/j.chest.2020.05.534

[7] Samaranayake CB Wright C Erigadoo S. A randomized controlled trial on optimal sampling sequence in radial guide sheath endobronchial ultrasound lung biopsy. J Bronchology Interv Pulmonol 2020;27:205–11. 32101915 10.1097/LBR.0000000000000651

[8] Sato M Shinohara Y Yanagiya M. Use of electromagnetic navigation bronchoscopy in virtual-assisted lung mapping: the effect of on-site adjustment. Gen Thorac Cardiovasc Surg 2019;67:1062–9. 31098868 10.1007/s11748-019-01137-z

[9] Sun F Xi J Zhan C. Ground glass opacities: imaging, pathology, and gene mutations. J Thorac Cardiovasc Surg 2018;156:808–13. 29753514 10.1016/j.jtcvs.2018.02.110

[10] Moon Y Sung SW Lee KY. Clinicopathological characteristics and prognosis of non-lepidic invasive adenocarcinoma presenting as ground glass opacity nodule. J Thorac Dis 2016;8:2562–70. 27747010 10.21037/jtd.2016.08.46 PMC5059258

[11] Kim HY Shim YM Lee KS. Persistent pulmonary nodular ground-glass opacity at thin-section CT: histopathologic comparisons. Radiology 2007;245:267–75. 17885195 10.1148/radiol.2451061682

[12] Moon Y Lee KY Park JK. The prognosis of invasive adenocarcinoma presenting as ground-glass opacity on chest computed tomography after sublobar resection. J Thorac Dis 2017;9:3782–92. 29268386 10.21037/jtd.2017.09.40 PMC5723887

[13] Acharya KV Unnikrishnan B Shenoy A. Utility of various bronchoscopic modalities in lung cancer diagnosis. Asian Pac J Cancer Prev 2017;18:1931–6. 28749623 10.22034/APJCP.2017.18.7.1931 PMC5648401

[14] Steinfort DP Leong TL Laska IF. Diagnostic utility and accuracy of rapid on-site evaluation of bronchoscopic brushings. Eur Respir J 2015;45:1653–60. 25537567 10.1183/09031936.00111314

[15] Biswas A Mehta HJ Sriram PS. Diagnostic yield of the virtual bronchoscopic navigation system guided sampling of peripheral lung lesions using ultrathin bronchoscope and protected bronchial brush. Turk Thorac J 2019;20:06–11. 10.5152/TurkThoracJ.2018.18030 PMC6340692 30664420

[16] Harris K Puchalski J Sterman D. Recent advances in bronchoscopic treatment of peripheral lung cancers. Chest 2017;151:674–85. 27292045 10.1016/j.chest.2016.05.025

[17] Ost DE Ernst A Lei X. Diagnostic yield and complications of bronchoscopy for peripheral lung lesions. Results of the AQuIRE registry. Am J Respir Crit Care Med 2016;193:68–77. 26367186 10.1164/rccm.201507-1332OC PMC4731617

